# Hard and Soft Tissue Facial Landmarks for Mandibular Angle Reduction: A Clinical Study

**DOI:** 10.3390/clinpract14050136

**Published:** 2024-08-27

**Authors:** Fei-Fan Tseng, Yu-Hsuan Li, Yuan-Wu Chen

**Affiliations:** 1 Department of Oral and Maxillofacial Surgery, Tri-Service General Hospital, No. 325, Section 2, Cheng-Kung Road, Neihu, Taipei 114, China; 2 School of Dentistry, National Defense Medical Center, No. 161, Section 6, Minquan E. Rd., Neihu, Taipei 114, China

**Keywords:** mandibular osteotomy, masseter muscle, multidetector computed tomography, Taiwan

## Abstract

Background: Square faces, which are influenced by genetic factors and structural features, are considered undesirable among the Asian population. Surgical interventions, such as mandibular angle reduction, aim to alter these characteristics, though complications may arise. We aimed to investigate the morphology of the mandibular angle and masseter muscle thickness using computed tomography (CT) and to analyze hard and soft tissue correlations to enhance surgical outcomes for patients with square faces. Methods: This retrospective clinical study included 100 Taiwanese patients aged 18–50 years. CT was used to analyze key clinical parameters, including bilateral mandibular width, mandibular divergence angle, ramus height, distance from the mandibular angle to the inferior alveolar nerve (IAN), and the thickness of the masseter muscle. Results: Significant correlations were noted between the patients’ physical height and weight, mandibular width, ramus height, masseter thickness, and distance from the angle to the IAN. Males exhibited a significantly longer and thicker ramus height (66.48 ± 4.28 mm), greater masseter thickness (15.46 ± 2.35 mm), and greater safety range for mandibular angle reduction surgery (18.35 ± 3.19 mm) (*p* < 0.00008). Significant correlations were observed among all parameters, except between mandibular width and gonial angle and the distance from the angle to the IAN and between mandibular divergence and masseter muscle thickness (*p* > 0.1). Conclusions: Our study highlighted the complex interplay among factors that contribute to square facial morphology. Careful preoperative assessments and customized surgical planning are essential for addressing this multifaceted clinical challenge.

## 1. Introduction

Square faces are often perceived as undesirable, masculine, and dominant among the Asian population. Square faces can be caused by genetic factors, the prominent width and gonial angle of the mandibular bone, masseter muscle hypertrophy, or an excessive volume of subcutaneous fat [1,2,3]. The lateral square face corresponds to a gonial angle less than the normal range (124.1 ± 5.67° in men and 125.59 ± 7.99° in women), approaching 90° [4]. The frontal square face is associated with an increased ratio between the bigonial and bizygomatic widths, although no specific ratio has been definitively reported [5]. A facial tapering ratio (bigonial width/bizygonial width) greater than 83% is considered to be indicative of a square face [6].

Various procedures, such as classical mandibular angle reduction, curved mandibular angle ostectomy, V-line ostectomy, lateral cortex splitting ostectomy, partial resection, botulinum toxin type A (BTA) injection of the masseter muscle, facial liposuction, and a lower facelift, have been utilized to address square facial features, with varying degrees of success [7,8,9]. Osteotomy or ostectomy cuts are typically performed using oscillating saws, reciprocating saws, or piezoelectric devices, and the bony edge is further smoothened using burs [7].

Complications such as numbness of the lower lip due to inferior alveolar nerve (IAN) injuries, swelling or hematoma, hemorrhage, infection, unfavorable fracture, sagging face, secondary angle formation, and facial asymmetry have been reported in 3.49–11.1% of cases [10,11]. Postoperative complications of BTA injections are temporarily observed in approximately 50% of patients with muscle weakness, reduced crunching power and bite force, or changes in facial expressions and facial asymmetry due to diffusion into the surrounding muscles [12,13].

The study may provide evidence of a correlation between the hard and soft tissue over the mandibular angle region. A comprehensive evaluation of ramus height, gonial angle, distance from the mandibular angle to the IAN, thickness of the masseter muscle, mandibular width, and divergence may be crucial for delicate facial profile analysis and surgical design. This approach is essential for achieving optimal outcomes in lower face contouring, regardless of whether a combination of hard and soft tissue revision is necessary [7,14,15]. In this study, we aimed to (1) investigate the correlations between soft tissue (thickness of the masseter muscle) and hard tissue (morphology of the mandible) parameters with patients’ body mass index (BMI) and (2) establish a safety zone for surgical correction of the square face in mandibular angle reduction to prevent IAN injury. We hypothesized that patients with square faces may have issues related to both bones and muscles simultaneously. We believe our study may provide a correlation between hard and soft tissue over the mandibular angle region. Thus, a better outcome may be achieved after the treatment.

## 2. Materials and Methods

### 2.1. Patients’ Selection

The sample size for the study was calculated to be approximately 100 cases (margin of error: 8.23%; confident level: 90%). This retrospective study included 100 patients who underwent digital measurements and assessments at Tri-Service General Hospital between January 2022 and November 2023. Participants were randomly selected from our database. The inclusion criteria were age of 18–50 years, absence of jaw developmental or pathological lesions, and no history of facial trauma or surgery. The exclusion criteria were osteoporosis or a history of antiresorptive medication use, bony metastasis, facial trauma, orthognathic surgery or other plastic surgery, IAN repositioning surgery, congenital craniofacial anomalies, or computed tomography (CT) images interfering with artifacts. This clinical study was approved by the Ethics Committee and Institutional Review Board of the Tri-Service General hospital. The need for informed consent was waived owing to the retrospective design of the study.

### 2.2. Anatomic Measurements

All measurements were examined by a single observer (F.F.T) who is specialized and certificated in Oral and Maxillofacial Surgery. To determine the intra-observer reliability, another observer (Y.H.L) evaluated 20 cases twice in a blinded manner. To assess the inter-observer reliability, the same cases were evaluated by another independent observer (Y.W.C) in a 1-month period. The reliability analysis was conducted using an intraclass correlation coefficient (ICC) test. All of the following parameters were measured using medical CT of mandible without contrast injection. All images were saved in the encrypted Digital Imaging and Communications in Medicine format.

**Figure 1:**
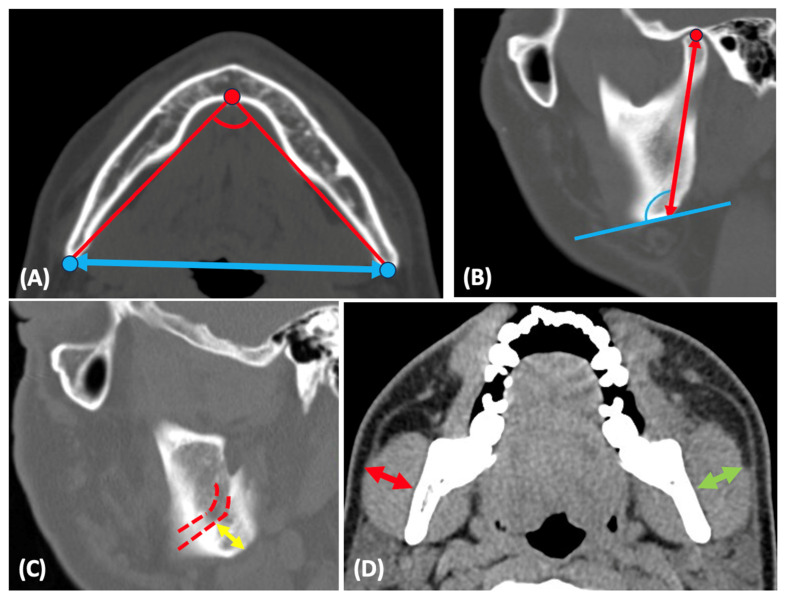
(**A**) Mandibular width (blue arrow) and divergence of the mandible (red line). Blue dots represent mandibular gonion and red dots represent menton. (**B**) Ramus height (red arrow) and gonial angle (blue angle). Red dots represent the condylion point. (**C**) Shortest distance from the mandibular angle to the inferior alveolar nerve (yellow arrow). Red dotted line represents the route of inferior alveolar nerve. (**D**) Masseter muscle thickness: right (red arrow) and left (green arrow) thickness measured in axial view.

Mandibular width: bigonial width (Figure 1A).

Mandibular divergence: The angle between bilateral gonial angle to menton (Figure 1A).

Ramus height: distance between the superior condylion (Cs) and gonion (Go) (Figure 1B).

Gonial angle: the angle between the posterior border of the ramus and the inferior border of the mandible (Figure 1B).

The shortest distance from the mandibular angle to IAN (Figure 1C).

The thickness of bilateral masseter muscle (Figure 1D).

### 2.3. Statistical Analysis

The patients’ data were recorded in Microsoft Excel. Statistical analyses were conducted using the IBM Statistical Package for the Social Sciences statistical software (version 24.0; IBM Corp., Armonk, NY, USA). Descriptive statistics (means and standard deviations) were calculated for all variables. Due to using continuous variables, an independent samples *t*-test was used to compare parameters between both sexes and sides. The Pearson correlation analysis was conducted to assess the existence of associations between various parameters. For all statistical analyses, the significance level was set at *p* < 0.05.

## 3. Results

### 3.1. Patients’ Demographics

The inter/intraobserver reliability analysis revealed a high level of reproducibility for all parameters in the mandibular angle region (*p* = 0.04). The participants’ characteristics are listed in Table 1. This clinical study included 100 patients with 200 sites, and the average age of the patients was 32.05 ± 9.16 years. The study cohort consisted of 50 male and 50 female individuals. No significant differences were noted in age between the sexes (*p* = 0.345) (Table 1).

### 3.2. Correlations between Patients’ Anthropometric Measures and Mandibular Hard and Soft Tissue Characteristics

A significant positive correlation was noted between height and mandibular width, ramus height, masseter thickness, and distance from the angle to the IAN (*p* < 0.0006). Similarly, a significant positive correlation was noted between weight and mandibular width, ramus height, and masseter thickness (*p* < 0.0004). However, only masseter muscle thickness showed a significant positive correlation with BMI (*p* = 0.00005) (Table 2).

### 3.3. Comparison of Clinical Parameters between Both Sexes and Sides

No significant differences were observed between the bilateral ramus height, gonial angle, masseter thickness, and distance from the mandibular angle to the IAN in both male and female participants (*p* > 0.12). However, male participants exhibited significantly longer and thicker rami, masseter thicknesses, and distances from the mandibular angle to the IAN than female participants (*p* < 0.00008). Additionally, the mandibular width was significantly wider in male participants than in female participants (*p* < 0.00001), while the mandibular divergence was significantly larger in female participants than in male participants (*p* = 0.041) (Table 3).

### 3.4. Correlations between Hard and Soft Tissue Parameters

Mandibular width was positively correlated with mandibular divergence, ramus height, and masseter thickness (*p* < 0.00004). Mandibular divergence was positively correlated with the gonial angle (*p* = 0.001) but negatively correlated with ramus height and distance from the angle to the IAN (*p* < 0.003). Ramus height was positively correlated with masseter thickness and distance from the mandibular angle to the IAN (*p* < 0.00001) but negatively correlated with the gonial angle (*p* = 0.042). Masseter muscle thickness was positively correlated with the distance from the mandibular angle to the IAN (*p* = 0.005) (Table 4).

## 4. Discussion

Our study revealed important insights into anatomical variations of the mandibular angle region, particularly regarding the relationship between soft and hard tissue, thereby providing valuable guidance for surgical planning and execution in mandibular angle reduction.

Mandibular growth and development are associated with several genetic, environmental, and demographic factors. Previous research has demonstrated that the vertical dimension of the posterior mandible, as represented by ramus height, is equally affected by both genetic and environmental stimuli [16]. During puberty, the annual growth rate of the mandible has been reported to be 2.16 mm for mandibular body length, 3.16 mm for ramus height, and 4.31 mm for the overall mandibular length, without significant sex-related or skeletal-type differences. This growth was statistically significant in the age range of 16–18 years [17,18]. Numerous studies have also consistently demonstrated statistically significant sex-based disparities in mandibular width and ramus height, with male individuals exhibiting larger dimensions than female individuals [19,20]. Conversely, the gonial angle exhibits insignificant sexual dimorphism, with values generally decreasing and stabilizing after the age of 21 years [21,22]. Our study findings corroborated these previously reported findings, further elucidating the strong correlations between mandibular width, divergence angle, ramus height, masseter thickness, and the distance from the mandibular angle to the IAN [23].

We explored the potential association between BMI and mandibular growth. Obesity affects both hard and soft tissues in terms of facial morphology by influencing bone metabolism and fat distribution [24]. Specifically, childhood obesity may accelerate the growth of facial bones, leading to increased facial skeletal dimensions, thereby resulting in elevated ramus height among overweight individuals [25]. Masseter muscle thickness has also been correlated with BMI, [26] which was also noted in our study. Facial growth has been linked to body height and the evidence suggests that facial growth may persist even after completion of skeletal maturation at 18 years of age [27]. In Turkey, facial bone height showed little correlation with body height [28]. Some studies have indicated that stature growth is related to posterior facial height, whereas others have proposed that mandibular ramus height could serve as an indicator of body height [29,30]. In this study, ramus height and mandibular width were correlated with body height and weight but not BMI.

Masseter muscle plays an important role in influencing the craniofacial morphology. Masseter muscle thickness is correlated with masticatory strength and function [31]. Moreover, masseter muscle thickness may affect facial morphology by increasing sagittal (anteroposterior) growth and limiting vertical growth of the jaws [32]. A thicker muscle may be associated with a shorter vertical facial height and may be positively correlated with ramus height [26,33]. The thickness of the masseter muscle may decrease as the mandible shows more prognathism [15]. Another report showed that the thickness of the masseter muscle was significantly positively associated with ramus height and thickness over the mandibular symphysis and negatively associated with the mandibular plane angle [33]. In our study, the relationship between hard and soft tissues was evident, with a significant positive correlation between masseter thickness, mandibular width, and ramus height. A study reported that excising the bilateral masseter muscle in immature rats led to underdevelopment of the mandible [34], suggesting that greater masseter muscle strength leads to enhanced mandibular growth.

Mandibular width, mandibular divergence, and the thickness of the ramus bone or masseter muscle represent the horizontal dimensions of the mandible. Some procedures, such as mandibular angle reduction and mandibular outer cortex split ostectomy, can improve the lateral and frontal square face, respectively [35]. In our study, we found that male patients had a greater safety distance, averaging 18.35 ± 3.19 mm, for mandibular angle reduction to spare IAN injury. This safety distance had a significant negative correlation with the gonial angle, indicating that patients with more lateral square faces and smaller gonial angles had more space for angle reduction. Our study also showed a positive correlation between mandibular width and masseter thickness and a negative correlation between masseter muscle thickness and gonial angle. These findings underscore that having a square face is due to a complex interplay between the bony and muscular components of the mandible. Therefore, preoperative evaluation of this region for customized design may be crucial.

This study has some limitations. First, all participants were recruited from a single medical institution in Taiwan and shared the same racial background. This may lead to a limited generalizability of our findings to patients of diverse ethnic backgrounds worldwide. Second, this study focused solely on evaluating a safe distance for mandibular angle reduction. The thickness of the bone over the angular region, which is crucial for the safe execution of the mandibular outer cortex split ostectomy, was not discussed in this study. This aspect will be analyzed and reported in a separate publication, and further data with a larger sample size are expected to support the findings from this study. This study is noteworthy for its analysis of the intricate relationships between the soft and hard tissues of the mandible. Diagnosis using both clinical findings and results of the radiographic examination of the mandibular divergence, degree of lateral protrusion over mandibular angle and hypertrophy of the masseter muscle should be evaluated in the future.

## 5. Conclusions

This study found several significant relationships between masseter muscle thickness and mandibular morphology, underscoring the importance of a comprehensive preoperative assessment and indicating that patients with square faces may have issues with both bone and soft tissue. Thus, a thorough examination and customized surgical design, tailored to address complex facial morphological concerns, may be essential for achieving optimal surgical outcomes. Future studies may include more patients to reduce the margin of error. Future studies may also compare soft tissue change between patients undergoing mandibular angle reduction, Botox injection, or both treatments.

## Figures and Tables

**Table 1:** Clinical characteristics of the patients (N = 100).

Variables	N (%)
Male	50 (50%)
Female	50 (50%)
Median age (years)	32.05 (9.16)
Median height (cm)	167.6 (8.73)
Median weight (kg)	65.82 (16.1)
Median BMI (kg/m^2^)	23.215 (4.62)

BMI, body mass index.

**Table 2:** Correlations between patients’ height and weight with measurement values of mandible and masseter muscle thickness.

Parameters	Height	Weight	BMI
Mandibular width	<0.00001	0.0004	0.06
Mandibular divergence	0.11	0.43	0.14
Ramus height	<0.00001	<0.00001	0.15
Gonial angle	0.55	0.97	0.64
Masseter thickness	0.00004	<0.00001	0.00005
Angle to IAN	0.0006	0.09	0.79

BMI, body mass index; IAN, inferior alveolar nerve.

**Table 3:** Comparison of hard and soft tissue morphology over mandibular angle between sides and sexes.

Parameters	Sex	Mean (SD)	Mean (SD)	*p*-Value	*p*-Value
Right ramus height (mm)	Male	66.55 (4.61)	66.48 (4.28)	0.88	<0.00001
Left ramus height (mm)		66.4 (4.56)			
Right ramus height (mm)	Female	59.7 (4.36)	59.35 (4.05)	0.42	
Left ramus height (mm)		59 (4.33)			
Right gonial angle (°)	Male	118.06 (7.54)	117.6 (7.02)	0.54	0.5
Left gonial angle (°)		117.14 (7.27)			
Right gonial angle (°)	Female	119.61 (6.92)	118.52 (6.66)	0.12	
Left gonial angle (°)		117.43 (7.08)			
Right masseter thickness (mm)	Male	15.23 (2.36)	15.46 (2.35)	0.35	<0.00001
Left masseter thickness (mm)		15.7 (2.61)			
Right masseter thickness (mm)	Female	12.67 (2.43)	12.67 (2.23)	0.99	
Left masseter thickness (mm)		12.68 (2.27)			
Right angle to IAN (mm)	Male	18.51 (3.26)	18.35 (3.19)	0.62	0.00008
Left angle to IAN (mm)		18.18 (3.44)			
Right angle to IAN (mm)	Female	16.07 (2.14)	16.14 (2.05)	0.74	
Left angle to IAN (mm)		16.21 (2.2)			
Mandibular width (mm)	Male	102.47 (6.47)		<0.00001	
	Female	96.25 (5.93)			
Mandibular divergence (°)	Male	72.34 (5.82)		0.041	
	Female	74.69 (5.57)			

IAN, inferior alveolar nerve; SD, standard deviation.

**Table 4:** Correlation between measurement values of the mandible and masseter muscle thickness.

Parameters	Mandibular Width	Mandibular Divergence	Ramus Height	Gonial Angle	Masseter Thickness	Angle to IAN
Mandibular width	-	<0.00001	<0.00001	0.57	0.00004	0.1
Mandibular divergence	<0.00001	-	0.003	0.001	0.16	0.00001
Ramus height	<0.00001	0.003	-	0.043	<0.00001	<0.00001
Gonial angle	0.57	0.001	0.043	-	0.042	<0.00001
Masseter thickness	0.00004	0.16	<0.00001	0.042	-	0.005
Angle to IAN	0.1	0.00001	<0.00001	<0.00001	0.005	-

IAN, inferior alveolar nerve.

## Data Availability

The datasets generated and/or analyzed during the current study are not publicly available to maintain privacy of the patients but can be obtained from the corresponding author on reasonable request.

## References

[1] Cole J.B., Manyama M., Larson J.R., Liberton D.K., Ferrara T.M., Riccardi S.L., Li M., Mio W., Klein O.D., Santorico S.A. (2017). Human facial shape and size heritability and genetic correlations. Genetics.

[2] Kundu N., Kothari R., Shah N., Sandhu S., Tripathy D.M., Galadari H., Gold M.H., Goldman M.P., Kassir M., Schepler H. (2022). Efficacy of botulinum toxin in masseter muscle hypertrophy for lower face contouring. J. Cosmet. Dermatol..

[3] Li J., Hsu Y., Khadka A., Hu J., Wang Q., Wang D. (2012). Surgical designs and techniques for mandibular contouring based on categorisation of square face with low gonial angle in orientals. J. Plast. Reconstr. Aesthetic Surg..

[4] Abdul Rehman S., Rizwan S., Shah Faisal S., Sheeraz Hussain S. (2020). Association of gonial angle on panoramic radiograph with the facial divergence on lateral cephalogram. J. Coll. Physicians Surg. Pak..

[5] Windhager S., Schaefer K., Fink B. (2011). Geometric morphometrics of male facial shape in relation to physical strength and perceived attractiveness, dominance, and masculinity. Am. J. Hum. Biol..

[6] Wu T.Y., Chou C.Y., Liang Y.M., Chang K.W., Wu C.H. (2021). A digital photograph study evaluating facial taperness and square face perception of Taiwanese females. J. Chin. Med. Assoc..

[7] Han M.D., Kwon T.G. (2023). Zygoma and mandibular angle reduction: Contouring surgery to correct the square face in Asians. Oral Maxillofac. Surg. Clin. N. Am..

[8] Cui J., Zhu S., Hu J., Li J., Luo E. (2008). The effect of different reduction mandibuloplasty types on lower face width and morphology. Aesthetic Plast. Surg..

[9] Bergeron L., Chen Y.R. (2009). The Asian face lift. Semin. Plast. Surg..

[10] Chen H., Sun J., Wang J. (2018). Reducing prominent mandibular angle osteotomy complications: 10-year retrospective review. Ann. Plast. Surg..

[11] Yoon E.S., Seo Y.S., Kang D.H., Koo S.H., Park S.H. (2006). Analysis of incidences and types of complications in mandibular angle ostectomy in Koreans. Ann. Plast. Surg..

[12] Kim N.H., Chung J.H., Park R.H., Park J.B. (2005). The use of botulinum toxin type A in aesthetic mandibular contouring. Plast. Reconstr. Surg..

[13] Kim N.H., Park R.H., Park J.B. (2010). Botulinum toxin type A for the treatment of hypertrophy of the masseter muscle. Plast. Reconstr. Surg..

[14] Andreishchev A.R., Nicot R., Ferri J. (2014). Mandibular angle resection and masticatory muscle hypertrophy—A technical note and morphological optimization. Rev. Stomatol. Chir. Maxillofac. Chir. Orale.

[15] Kim T.H., Kim C.H. (2020). Correlation between mandibular morphology and masticatory muscle thickness in normal occlusion and mandibular prognathism. J. Korean Assoc. Oral Maxillofac. Surg..

[16] Hersberger-Zurfluh M.A., Motro M., Kantarci A., Will L.A., Eliades T., Papageorgiou S.N. (2024). Genetic and environmental impact on mandibular growth in mono- and dizygotic twins during adolescence: A retrospective cohort study. Int. Orthod..

[17] Gomes A.S., Lima E.M. (2006). Mandibular growth during adolescence. Angle Orthod..

[18] Sharma P., Arora A., Valiathan A. (2014). Age changes of jaws and soft tissue profile. Sci. World J..

[19] Sairam V., Geethamalika M.V., Kumar P.B., Naresh G., Raju G.P. (2016). Determination of sexual dimorphism in humans by measurements of mandible on digital panoramic radiograph. Contemp. Clin. Dent..

[20] Olayemi A.B. (2011). Assessment and determination of human mandibular and dental arch profiles in subjects with lower third molar impaction in Port Harcourt, Nigeria. Ann. Maxillofac. Surg..

[21] Larrazabal-Moron C., Sanchis-Gimeno J.A. (2018). Gonial angle growth patterns according to age and gender. Ann. Anat..

[22] Bulut O., Freudenstein N., Hekimoglu B., Gurcan S. (2019). Dilemma of gonial angle in sex determination: Sexually dimorphic or not?. Am. J. Forensic Med. Pathol..

[23] Coquerelle M., Bookstein F.L., Braga J., Halazonetis D.J., Weber G.W., Mitteroecker P. (2011). Sexual dimorphism of the human mandible and its association with dental development. Am. J. Phys. Anthr..

[24] López-Gómez J.J., Pérez Castrillón J.L., de Luis Román D.A. (2016). Impact of obesity on bone metabolism. Endocrinol. Nutr..

[25] Gordon L.A., Miller S.F., Caplin J., Galang-Boquiren M.T., Alrayyes S., Nicholas C.L. (2021). Childhood obesity may accelerate timing of human facial growth. Arch. Oral Biol..

[26] Satiroğlu F., Arun T., Işik F. (2005). Comparative data on facial morphology and muscle thickness using ultrasonography. Eur. J. Orthod..

[27] Luzi V., Colangelo G., Scotti L. (1983). Correlation between facial height and body height during the prepubertal period. Dent. Cadmos.

[28] Pelin C., Zağyapan R., Yazici C., Kürkçüoğlu A. (2010). Body height estimation from head and face dimensions: A different method. J. Forensic Sci..

[29] Moore R.N., Moyer B.A., DuBois L.M. (1990). Skeletal maturation and craniofacial growth. Am. J. Orthod. Dentofac. Orthop..

[30] Van der Beek M.C., Hoeksma J.B., Prahl-Andersen B. (1996). Vertical facial growth and statural growth in girls: A longitudinal comparison. Eur. J. Orthod..

[31] Kiliaridis S., Georgiakaki I., Katsaros C. (2003). Masseter muscle thickness and maxillary dental arch width. Eur. J. Orthod..

[32] Tircoveluri S., Singh J.R., Rayapudi N., Karra A., Begum M., Challa P. (2013). Correlation of masseter muscle thickness and intermolar width—An ultrasonography study. J. Int. Oral Health.

[33] Kubota M., Nakano H., Sanjo I., Satoh K., Sanjo T., Kamegai T., Ishikawa F. (1998). Maxillofacial morphology and masseter muscle thickness in adults. Eur. J. Orthod..

[34] Yonemitsu I., Muramoto T., Soma K. (2007). The influence of masseter activity on rat mandibular growth. Arch. Oral Biol..

[35] Li X., Hsu Y., Hu J., Khadka A., Chen T., Li J. (2013). Comprehensive consideration and design for treatment of square face. J. Oral Maxillofac. Surg..

